# TGFβ1 regulates HGF‐induced cell migration and hepatocyte growth factor receptor MET expression via C‐ets‐1 and miR‐128‐3p in basal‐like breast cancer

**DOI:** 10.1002/1878-0261.12355

**Published:** 2018-07-30

**Authors:** Christian Breunig, Nese Erdem, Alexander Bott, Julia F. Greiwe, Eileen Reinz, Stephan Bernhardt, Chiara Giacomelli, Astrid Wachter, Eva J. Kanthelhardt, Tim Beißbarth, Martina Vetter, Stefan Wiemann

**Affiliations:** ^1^ Division of Molecular Genome Analysis German Cancer Research Center (DKFZ) Heidelberg Germany; ^2^ Department of Medical Statistics University Medical Center Göttingen Germany; ^3^ Department of Gynecology Martin‐Luther‐University Halle Wittenberg Germany

**Keywords:** ETS1, MET, microRNA‐128‐3p, TGFBR2, TGFβ, triple‐negative breast cancer

## Abstract

Breast cancer is the most common cancer in women worldwide. The tumor microenvironment contributes to tumor progression by inducing cell dissemination from the primary tumor and metastasis. TGFβ signaling is involved in breast cancer progression and is specifically elevated during metastatic transformation in aggressive breast cancer. In this study, we performed genomewide correlation analysis of *TGFBR2* expression in a panel of 51 breast cancer cell lines and identified that *MET* is coregulated with *TGFBR2*. This correlation was confirmed at the protein level in breast cancer cell lines and human tumor tissues. Flow cytometric analysis of luminal and basal‐like breast cancer cell lines and examination of 801 tumor specimens from a prospective cohort of breast cancer patients using reverse phase protein arrays revealed that expression of TGFBR2 and MET is increased in basal‐like breast cancer cell lines, as well as in triple‐negative breast cancer tumor tissues, compared to other subtypes. Using real‐time cell analysis technology, we demonstrated that TGFβ1 triggered hepatocyte growth factor (HGF)‐induced and MET‐dependent migration *in vitro*. Bioinformatic analysis predicted that TGFβ1 induces expression of C‐ets‐1 as a candidate transcription factor regulating *MET* expression. Indeed, TGFβ1‐induced expression of *ETS1* and breast cancer cell migration was blocked by knockdown of *ETS1*. Further, we identified that *MET* is a direct target of miR‐128‐3p and that this miRNA is negatively regulated by TGFβ1. Overexpression of miR‐128‐3p reduced *MET* expression and abrogated HGF‐induced cell migration of invasive breast cancer cells. In conclusion, we have identified that TGFβ1 regulates HGF‐induced and MET‐mediated cell migration, through positive regulation of C‐ets‐1 and negative regulation of miR‐128‐3p expression in basal‐like breast cancer cell lines and in triple‐negative breast cancer tissue.

**Abbreviations**
CAF: cancer‐associated fibroblast
ER: estrogen receptor
HGF: hepatocyte growth factor
RPPA: reverse phase protein array
TGFBR2: TGFβ receptor type‐2
TGFβ1: transforming growth factor beta 1
TNBC: triple‐negative breast cancer

## 1 Introduction

Breast cancer is the most common cancer in women worldwide with over 1.5 million new breast cancer cases diagnosed every year, which makes up to 30% of all cancers (Siegel *et al*., 2017). It is a heterogeneous disease, and the appearance of drug resistance and formation of metastasis are leading causes of mortality (Di Cosimo and Baselga, 2010; Weigelt *et al*., 2005). The tumor microenvironment contributes to tumor progression by inducing cell dissemination from the primary tumor and metastasis. Various cytokines and growth factors are secreted by cells of the tumor microenvironment and are involved in processes that potently promote tumor growth as well as metastasis formation (Breunig *et al*., 2014; Korkaya *et al*., 2011).

Transforming growth factor beta 1 (TGFβ1) is one major player in this process and has been described as a double edged sword in breast cancer (Bierie and Moses, 2006). While TGFβ1 inhibits cell growth at an early stage of carcinogenesis, it supports metastasis formation in late‐stage cancer (Imamura *et al*., 2012). We have previously shown that TGFβ signaling and TGFβ receptor type‐2 (TGFBR2) seem to have a tumor promoting role in human estrogen receptor (ER)‐negative breast cancer (Keklikoglou *et al*., 2012). In addition, clinical studies in ER‐negative breast tumors indicated a correlation of elevated TGFBR2 levels with shorter overall breast cancer patient survival (Buck *et al*., 2004). However, the molecular mechanisms of TGFβ signaling and elevated TGFBR2 expression are likely diverse and have not been fully elucidated.

Transcriptional and post‐transcriptional regulations of gene and protein expression are well‐described mechanisms of metastasis regulation. Transcriptional regulation involves transcription factors, which are key regulators for gene expression. Several transcription factors are deregulated in human carcinomas and vastly contribute to tumor progression (Lee and Young, 2013; Willis *et al*., 2015). Due to this deregulation, transcription factors such as ER alpha (ERα) in breast cancer represent attractive targets for cancer therapy (Darnell, 2002). miRNAs regulate signaling pathways at the post‐transcriptional level and are frequently deregulated in breast cancer. It has been shown that aberrantly expressed miRNAs promote cancer development, metastasis formation, and potently induce drug resistance in both tumor cells and cells of the tumor microenvironment (Bott *et al*., 2017; Breunig *et al*., 2017; Dvinge *et al*., 2013; Tang *et al*., 2012; Zhu *et al*., 2011). In a previous study, we have identified a tumor‐suppressive function of the miR‐520/miR373 family by targeting TGFBR2 in breast cancer cells (Keklikoglou *et al*., 2012).

The hepatocyte growth factor (HGF) is another major player promoting tumor growth as well as metastasis formation. HGF is secreted by stromal cells such as cancer‐associated fibroblast (CAF) in the tumor microenvironment (Casbas‐Hernandez *et al*., 2013). Upon binding of HGF to its receptor MET, it induces the activation of downstream FAK, MAPK, and PI3K/Akt signaling (Birchmeier *et al*., 2003) and thereby leads to the regulation of a wide range of cellular processes such as metastasis formation in breast cancer cells (Ho‐Yen *et al*., 2015). MET expression is a prognostic factor in breast carcinoma, and high levels of MET have been shown to correlate with poor survival of patients with breast cancer (Zhao *et al*., 2017). Expression of MET is elevated in basal‐like tumors and inflammatory breast carcinoma (Garcia *et al*., 2007; Ponzo and Park, 2010); however, it is still not fully understood how MET is regulated in breast cancer cells.

In this study, we performed a genomewide correlation analysis of *TGFBR2* in a panel of 51 breast cancer cell lines (Riaz *et al*., 2013) to identify genes which are coregulated with *TGFBR2* and to test their impact on cell migration. *MET* was one of the top positively correlated genes with *TGFBR2* in these breast cancer cell lines. Clinical significance of our *in vitro* findings was validated by analyzing 801 breast cancer tissue samples of a multicenter prospective study (NCT01592825). There, the same correlation was observed also at the protein level. TGFBR2 and MET were both significantly stronger expressed in triple‐negative breast tumors (TNBC) than in luminal‐like specimen. We identified and characterized the transcription factor C‐ets‐1 and miR‐128‐3p as regulators of MET expression that are both driven by the TGFβ signaling pathway *in vitro*. Inhibition of C‐ets‐1 and overexpression of miR‐128‐3p significantly repressed MET expression and thereby inhibited the HGF/MET signaling pathway as well as migration toward HGF.

## 2 Materials and methods

### 2.1 Cell culture and cell lines

Cell lines MDA‐MB‐231 (HTB‐26), MCF‐7 (HTB‐22), T47D (HTB‐133), HCC1143 (CRL‐2321), BT549 (HTB‐122), UACC812 (CRL‐1897), MDA‐MB‐468 (HTB‐132), BT474 (HTB‐20), HCC1954 (CRL‐2339), SKBR‐3 (HTB‐30), HS578T (HTB‐126), and MCF10A (CRL‐10317) were obtained from American Type Culture Collection (LGC Standards GmbH, Wesel, Germany). MDA‐MB‐231 cells were maintained in Leibovitz's L‐15 (Life Technologies, Carlsbad, CA, USA) medium [10% FBS, 1% l‐glutamine, 1% nonessential amino acids (NEAA), 50 units·mL^−1^ penicillin, 50 μg·mL^−1^ streptomycin sulfate (all Life Technologies)]. MCF‐7 cells were cultured in MEM (10% FBS, 1% NEAA, 0.01 mg·mL^−1^ bovine insulin (Sigma‐Aldrich, St. Louis, MO, USA), 50 units·mL^−1^ penicillin, 50 μg·mL^−1^ streptomycin sulfate). T47D cells were cultured in RPMI (Life Technologies) medium (10% FBS, 1% NEAA, 50 units·mL^−1^ penicillin, 50 μg·mL^−1^ streptomycin sulfate), and MDA‐MB‐468, BT549, UACC812, HCC1954, and HCC1143 cells were cultured in RPMI medium (10% FBS, 50 units·mL^−1^ penicillin, 50 μg·mL^−1^ streptomycin sulfate). BT474 cells were cultured in DMEM (Life Technologies) (10% FBS). HS5578T cells were cultured in DMEM (10% FBS, 0.01 mg·mL^−1^ bovine insulin). SKBR‐3 cells were cultured in McCoy's 5A (Life Technologies) (10% FBS). MCF10A cells were cultured in DMEM F12 medium (5% horse serum (Life Technology), 20 ng·mL^−1^ EGF (BD Biosciences, Franklin Lakes, NJ, USA), 0.5 μg·mL^−1^ hydrocortisone (Sigma‐Aldrich), 100 ng·mL^−1^ cholera toxin (Sigma‐Aldrich), 0.01 mg·mL^−1^ bovine insulin (Sigma‐Aldrich), 50 units·mL^−1^ penicillin and 50 μg·mL^−1^ streptomycin sulfate). Authentication as well as contamination tests of all cell lines was performed at Multiplexion GmbH (Heidelberg, Germany).

### 2.2 Transfections and reagents

Transfections of siRNA, miRNAs, and luciferase vectors were performed using Lipofectamine 2000 or Lipofectamine RNAiMax (both Invitrogen, Carlsbad, CA, USA) according to manufacturer's instructions. ON TARGETplus siRNAs targeting MET, TGFBR2, and C‐ets‐1 were from Dharmacon (Lafayette, CO, USA), where three to four individual siRNAs were used as single siRNA or pooled (Table S1). ON TARGETplus nontargeting siRNA pool (Dharmacon) was used as control. miRIDIAN miRNA mimics miR‐128‐3p (5′ UCACAGUGAACCGGUCUCUUU 3′), miR‐128‐3p hairpin inhibitors, and negative controls (miRNA control and inhibitor control) were obtained from Dharmacon. siRNAs, miRNA mimics, and miRNA inhibitors were used at a final concentration of 30 or 50 nm.

For HGF and TGFβ treatment, cells were seeded in six‐well plates and treated with 75 ng·mL^−1^ HGF and 10 ng·mL^−1^ TGFβ1, respectively (both R&D systems, Minneapolis, MN, USA). TGFBR2 inhibitor ITD1 (Tocris, Bristol, UK) was used at a concentration of 2.5 μm. Cells were treated with inhibitors 1 h prior addition of HGF or TGFβ1. For MET knockdown experiments, MCF10A or HCC1143 cell lines were transfected with single or pooled siMET or siRNA control 24 h prior addition of TGFβ1.

### 2.3 Luciferase reporter assays

To validate direct targeting of miR‐128‐3p, psiCHECK2 vectors (Promega, Fitchburg, WI, USA), containing the respective 3′UTRs, were cotransfected with mimic miRNAs in MCF‐7 cells. Forty‐eight hours after transfection, Renilla and firefly luciferase activities were determined using a luminometer (Tecan, Männedorf, Switzerland). Mutations within the predicted target site of MET 3′‐UTRs were generated by site‐directed mutagenesis using QuikChange II Site‐Directed Mutagenesis Kit (Agilent Technologies, Santa Clara, CA, USA) according to the manufacturer's instructions.

### 2.4 Antibodies, immunoblotting, and flow cytometry

For western blotting, cells were lysed in ice cold M‐PER lysis buffer (Thermo‐Fisher Scientific, Waltham, MA, USA) containing NaF (Th. Geyer), Na_3_VO_4_ (Sigma‐Aldrich), and protease inhibitor Complete Mini and phosphatase inhibitor PhosSTOP (both Roche, Basel, Switzerland). Protein concentrations were determined by BCA Protein Assay Reagent Kit (Thermo‐Fisher Scientific), and proteins were denatured with 4× Roti Load (Carl Roth, Karlsruhe, Germany) at 95 °C for 5 min. Depending on the size, proteins were separated by 10 and 15% SDS/PAGE, blotted onto a PVDF membrane Immobilon‐FL (Merck Millipore, Darmstadt, Germany), and exposed to primary antibodies. The following antibodies were used: MET (clone L41G3; CST #3148), phospho‐MET (clone D26; CST #3077), C‐ets‐1 (clone D8O8A; CST # 14069), β‐actin (polyclonal; Sigma‐Aldrich #20‐33), and β‐actin (clone C4; MP Biochemicals #691001). Blots were probed with goat anti‐rabbit or goat anti‐mouse IgG (H+L) IRDye^®^680‐ or IRDye^®^780‐conjugated secondary antibodies (Thermo‐Fisher Scientific), and bands were visualized using an Odyssey scanner (LI‐COR, Lincoln, NE, USA). Primary antibodies were used at a 1 : 1000 dilution and secondary antibodies at a 1 : 10 000 dilution.

For flow cytometry, cells were detached using Accutase (Life Technology), washed, and resuspended into FACS buffer (BD Biosciences). Cells were stained for MET and TGFBR2 expression using 5 μg·mL^−1^ anti‐MET (clone 95106; R&D systems #MAB3582) or anti‐TGFBR2 primary antibodies (clone MM0056‐4F14; Abcam #ab78419, Cambridge, UK). A mouse IgG isotype was used as a control (clone UPC‐10; Sigma‐Aldrich #M5409). Goat anti‐mouse IgG/IgM antibody coated with FITC was used as a secondary antibody in a 1 : 100 dilution (polyclonal; BD Biosciences #555988). Specificity of MET and TGFBR2 primary antibodies were shown with knockdown experiments using siRNA against MET and TGFBR2, respectively (Fig. S1). Cells were analyzed using FACSCalibur (BD Biosciences) and flowjo (Treestar, Ashland, OR, USA).

### 2.5 Patient samples

Tissue lysates from fresh‐frozen breast cancer patient samples were derived from the multicenter prospective PiA study (NCT01592825) as previously described (Bernhardt *et al*., 2017). Breast cancer subtypes were defined to histopathological characteristics such as receptor status and grading according to the St. Gallen classification and by von Minckwitz and colleagues (Goldhirsch *et al*., 2013; von Minckwitz *et al*., 2012).

### 2.6 Ethics approval and consent to participate

Our study is in accordance with the Declaration of Helsinki. Institutional review board approval by the Ethics Committee of the Medical Faculty of the Martin‐Luther‐University Halle‐Wittenberg was received, and informed consent was obtained from all patients.

### 2.7 Reverse phase protein array (RPPA)

The method used in this study has been described previously (Loebke *et al*., 2007). Briefly, RPPA was performed on 2 μg·μL^−1^ of protein, lysed with tissue protein extraction reagent (50 mm Tris, pH 8.5, 138 mm NaCl, 2.7 mm KCl, 1% Triton X‐100). Lysed protein was mixed with 4× SDS sample buffer (10% glycerol, 4% SDS, 10 mm DTT, 125 mm Tris/HCl, pH 6.8) and denaturated at 95 °C for 5 min. Protein samples were printed onto nitrocellulose‐coated glass slides (Oncyte Avid; Grace‐Biolabs, Bend, OR, USA) with a Aushon 2470 contact spotter (Aushon BioSystems, Billerica, MA, USA) and stored at −20 °C until further use.

Slides were blocked with blocking buffer (Rockland Immunochemicals) in TBS (50%, v/v) containing 5 mm NaF and 1 mm Na_3_VO_4_ for 2 h at room temperature. Slides were incubated at 4 °C over night with primary antibodies diluted at 1 : 300 and subsequently washed four times for 5 min in 1× TBS with 0.1% Tween‐20. The antibodies used were MET (CST 3148; Cell Signaling Technology, Danvers, MA, USA) and TGFBR2 (Santa Cruz Biotechnology, #sc‐17799, Heidelberg, Germany). Next, slides were incubated for 1 h with infrared‐labeled secondary antibody, Alexa Fluor 680 F(ab′)2 fragments of goat anti‐mouse IgG (Life Technologies) diluted at 1 : 8000 in 1× TBS with 0.1% Tween‐20. Washing steps were performed as described above. All washing and incubation steps were carried out at room temperature with gentle shaking. Finally, slides were rinsed in water and air‐dried at room temperature. Slides were scanned using the Odyssey Scanner (LI‐COR, Biosciences) with 21 μm resolution. TIFF images of all slides were obtained at an excitation wavelength of 685 nm.

Signal intensities of each individual spot were quantified using genepixpro 7.0 (Molecular Devices, LLC, San José, CA, USA). The R‐package RPPanalyzer (version 1.4) was used for data preprocessing and quality control. Data were log2‐transformed subsequently. For visualizing target expression levels in box plots, RPPA data were normalized by scaling into the range between 0 and 1.

### 2.8 RNA isolation and quantitative real‐time PCR

Total RNA and miRNA were isolated from cells using miRNeasy Mini Kit (Qiagen, Hilden, Germany) according to the manufacturer's instructions. For mRNA, cDNA synthesis was carried out with the Revert Aid H Minus First Strand cDNA Synthesis Kit (Fermentas, Waltham, MA, USA). The quantitative RT‐PCRs (qRT‐PCRs) for target genes were performed using ABI Prism 7900HT Sequence Detection System (Applied Biosystems, Waltham, MA, USA), using probes from the Universal Probe Library (Roche) (Table S2). The housekeeping genes ACTB and TFRC were used for normalization of mRNA analysis. For miRNAs, the TaqMan microRNA reverse transcription kit and TaqMan gene‐specific microRNA assays (Applied Biosystems) were used. For the quantitative RT‐PCRs (qRT‐PCRs), RNU44 and RNU48 were used as housekeeping controls. Data were acquired using a HT‐7900 TaqMan instrument (Applied Biosystems) and analyzed with the ΔΔCT algorithm (ddCt; Bioconductor).

### 2.9 RTCA migration assay

RTCA (real‐time cell analyzer) cell migration assays were performed as previously described (Breunig *et al*., 2014). For RTCA migration experiments, transfections and/or treatment with inhibitors and TGFβ1 were performed as described above. Twenty‐four hours prior experiment cells were starved with cell growth medium without FBS. Then, 100 000 cells were seeded in the upper chamber of the RTCA CIM‐plate 16 (Roche) in starvation medium. The lower chamber was filled with starvation medium with or without 75 ng·mL^−1^ HGF (R&D Systems). CIM‐plate was inserted in the xCELLigence machine, and migration was measured every 15 min up to 30 h depending on the cell line.

### 2.10 Analysis of cell lines and breast cancer tissue and statistical analysis

A dataset containing mRNA expression of 51 breast cancer cells (Riaz *et al*., 2013) was used for mRNA expression analysis of TGFBR2 and MET. Cell lines were classified into luminal and basal according to their molecular characteristics (Neve *et al*., 2006). The microarray data were obtained from the Gene Expression Omnibus data repository (GEO: GSE41313). In addition, this dataset was used to calculate the Pearson product–moment correlation coefficient of all genes toward TGFBR2 and MET. An additional dataset containing mRNA expression data of 48 breast cancer cell lines (Kao *et al*., 2009) was used for validation. This microarray data were obtained from the Gene Expression Omnibus data repository (GEO: GSE15376). *TGFBR2* and *MET* gene expression data from the NCI‐60 panel, Sanger cell line panel as well as the TCGA datasets were obtained from the R2: Genomics Analysis and Visualization Platform (http://r2.amc.nl). Two datasets which included mRNA and miRNA expression data for human primary breast tumors were obtained from the NCBI GEO database (GEO: GSE19783) and from the METABRIC dataset (EGAC01000000010) were used (Curtis *et al*., 2012). Potential transcription factor binding to the promoter of MET was analyzed using the Transcriptional Regulatory Element Database (http://rulai.cshl.edu/TRED). Correlations and statistical analyses were carried out with graphpad software (GraphPad software Inc., La Jolla, CA, USA) to generate Kaplan–Meier curves and boxplots. Unless otherwise stated, all *P*‐values were calculated by means of a two‐sided *t*‐test where *P*‐values < 0.05 were considered as significant.

## 3 Results

### 3.1 TGFBR2 is higher expressed in basal‐like breast cancer and correlates with hepatocyte growth factor receptor expression

**Figure 1:**
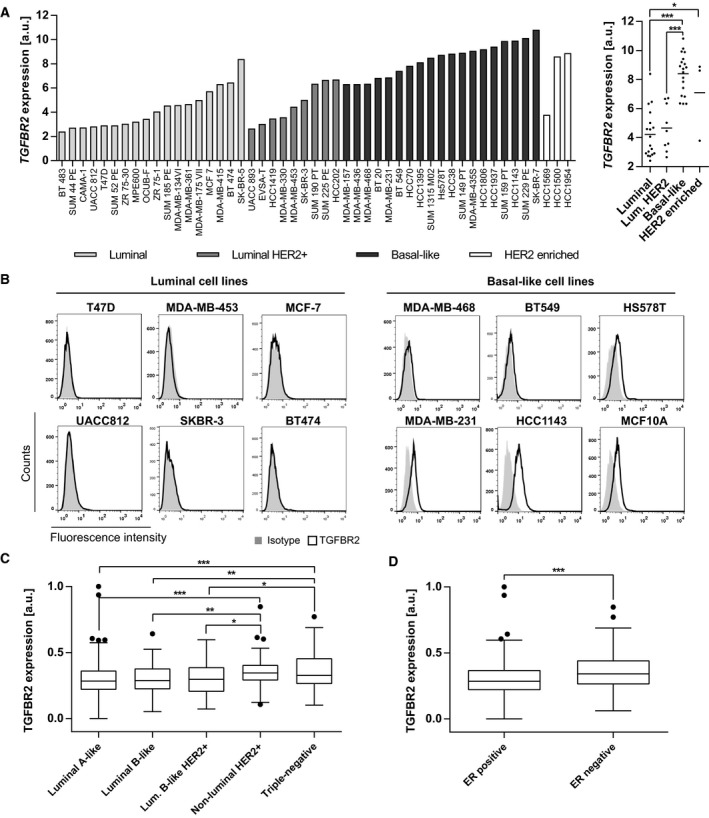
TGFBR2 is higher expressed in basal‐like breast cancer cell lines and triple‐negative breast cancer tissue. (A) Quantitative PCR for *TGFBR2* mRNA expression in breast cancer cell lines using breast cancer cell line microarray database for *TGFBR2* (Riaz *et al*., 2013) (luminal *n* = 18; luminal HER2 *n* = 9; basal‐like *n* = 19; HER2 enriched *n* = 3; Student's unpaired *t*‐test). (B) Flow cytometry analysis for TGFBR2 of breast cancer cell lines of different subtypes and the fibrocystic cell line with the basal‐like genotype MCF10A. (C) TGFBR2 protein expression in breast cancer patient subtypes luminal A‐like (*n* = 510), luminal B‐like (*n* = 104), luminal B‐like HER2+ (*n* = 74), nonluminal HER2+ (*n* = 36), and triple‐negative (*n* = 77) measured by RPPA. (D) TGFBR2 protein expression in ER‐negative (ER neg.; *n* = 120) and ER‐positive (ER pos.: *n* = 681) breast cancer specimens analyzed by RPPA. Data are expressed as mean for (A) and represented in a box and whiskers plot (in the style of Tukey) for (C) and (D). Key: *P < 0.05; **P < 0.01; ***P < 0.001.

We recently described a tumor promoting role of TGFβ signaling and TGFBR2 in human ER‐negative breast cancer (Keklikoglou *et al*., 2012). To further elaborate on the function of TGFBR2 within subtypes of human breast cancer, we initially analyzed a gene expression dataset from 51 breast cancer cell lines (Riaz *et al*., 2013). There, we identified *TGFBR2* as being higher expressed in basal‐like compared to luminal as well as higher in ER‐negative compared to ER‐positive breast cancer cell lines (Figs 1A and S2). To validate these findings, we measured surface expression of TGFBR2 at the protein level in several breast cancer cell lines confirming elevated expression in basal‐like compared to luminal cell lines (Fig. 1B). Next, we measured TGFBR2 expression at the protein level in a set of 801 tissue samples of a prospective breast cancer cohort to investigate on TGFBR2 expression in different breast cancer subtypes *in vivo*. Here, we detected higher TGFBR2 expression in triple‐negative tumors compared to luminal‐like tumors (Fig. 1C) as well as in ER‐negative compared to ER‐positive breast tumor tissue (Fig. 1D).

**Figure 2:**
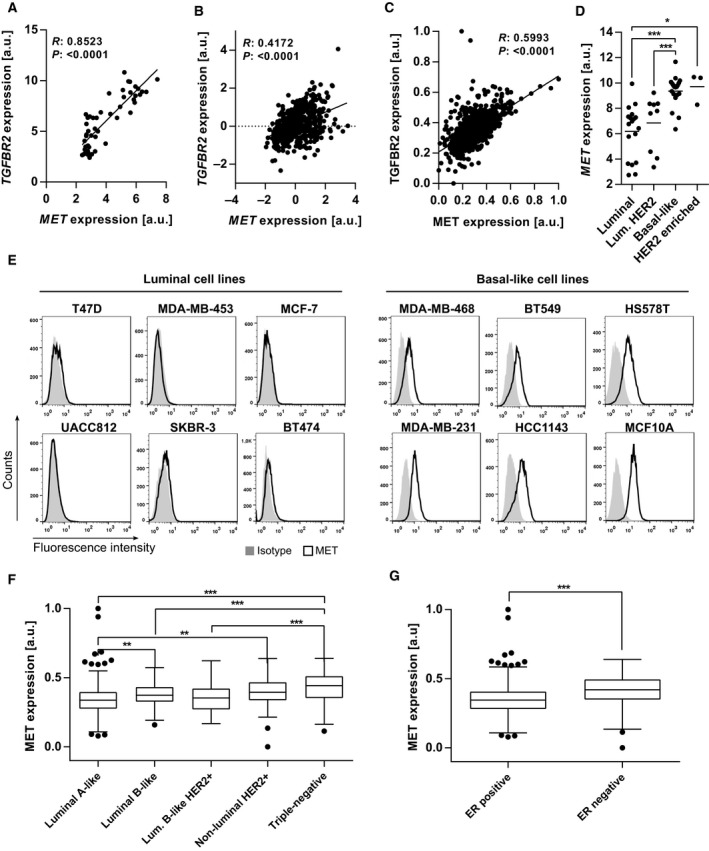
MET correlates with TGFBR2 expression and is expressed at higher levels in basal‐like breast cancer cell lines and triple‐negative breast cancer tissue. (A, B) Correlation analysis of *MET* and *TGFBR2* mRNA expression in (A) breast cancer cell lines with each data point representing a different breast cancer cell line (Pearson's correlation, breast cancer cell line microarray database, *n* = 51) (Riaz *et al*., 2013), (B) breast cancer patients with each data point representing an individual sample [Pearson's correlation (*R*); TCGA breast cancer dataset, *n* = 547]. (C) Correlation of MET and TGFBR2 protein expression in breast cancer patients with each data point representing an individual sample [Pearson's correlation (*R*); *n* = 801]. Protein expression was measured with RPPA. (D) Quantitative PCR mRNA expression analysis in breast cancer cell lines using breast cancer cell line microarray database (Riaz *et al*., 2013). (E) Flow cytometry analysis of breast cancer cell lines of luminal and basal‐like breast cancer subtypes and the fibrocystic cell line with the basal‐like genotype MCF10A. (F) MET protein expression in breast cancer patient subtypes luminal A‐like (*n* = 510), luminal B‐like (*n* = 104), luminal B‐like HER2+ (*n* = 74), nonluminal HER2+ (*n* = 36), and triple‐negative (*n* = 77) measured by RPPA. (G) MET protein expression in ER‐negative (ER neg.; *n* = 120) and ER‐positive (ER pos.; *n* = 681) breast cancer specimens analyzed by RPPA. Data are expressed as mean for (D) and represented in a box and whiskers plot (in the style of Tukey) for (F) and (G). Key: *P < 0.05; **P < 0.01; ***P < 0.001.

We hypothesized that genes which are coexpressed with TGFBR2 could be involved in TGFβ‐mediated progression of breast cancer. Therefore, a correlation analysis using gene expression data from a 51 cell line panel (Riaz *et al*., 2013) was performed to identify genes that are positively coexpressed with *TGFBR2* in tumor cells. The hepatocyte growth factor receptor (*MET*) was among the top significant genes positively correlating with *TGFBR2* expression (Fig. 2A and Table S3), which could be validated using an independent dataset of breast cancer cell lines (Fig. S3A) (Kao *et al*., 2009). Positive correlation of *MET* with *TGFBR2* gene expression was also observed in breast cancer tissue using the breast cancer TCGA dataset (Fig. 2B) and, at the protein level, in 801 breast cancer specimens (Fig. 2C). Besides breast cancer, a putative relationship between *MET* and *TGFBR2* expression was observed also in cell lines from other tumor entities using the NCI‐60 as well as the 789 cell line panels of the NCI and the Sanger Institute, respectively (Fig. S3B, C). These correlations could be validated by analyzing publicly available patient datasets. *MET* and *TGFBR2* gene expressions were found to positively correlate in several other tumor entities, such as prostate adenocarcinoma, thymoma, glioblastoma, head and neck squamous cell carcinoma, testicular germ cell tumors, and esophageal carcinoma (Fig. S3D–I).

Next, we investigated whether the expression of MET is related to specific breast cancer subtypes and therefore checked its differential expression in cell lines of different breast cancer subtypes (Riaz *et al*., 2013). This analysis revealed that *MET* is higher expressed in basal‐like compared to luminal as well as in ER‐negative compared to ER‐positive breast cancer cell lines (Figs 2D and S4A). To validate these findings, we analyzed surface expression of MET by flow cytometry. Luminal breast cancer cell lines MCF‐7, T47D, and MDA‐MB‐453 as well as the luminal HER2+ breast cancer cell lines BT474 and SKBR‐3 had very little surface expression of MET. In contrast, MET was expressed at substantial levels in basal‐like breast cancer cell lines MDA‐MB‐468, BT549, HS578T, MDA‐MB‐231, and HCC1143, as well as the HER2‐enriched cell line HCC1954 and in the immortalized breast epithelial cell line MCF10A (Figs 2E and S4B). Protein expression analysis revealed higher MET expression in triple‐negative compared to luminal‐like breast tumors in a set of 801 breast cancer tissue samples (Fig. 2F). As for the 51 breast cancer cell lines, MET was higher expressed in breast tumors lacking ER expression than in samples with ER expression (Fig. 2G).

### 3.2 TGFβ1 induces cell migration via upregulation of MET

**Figure 3:**
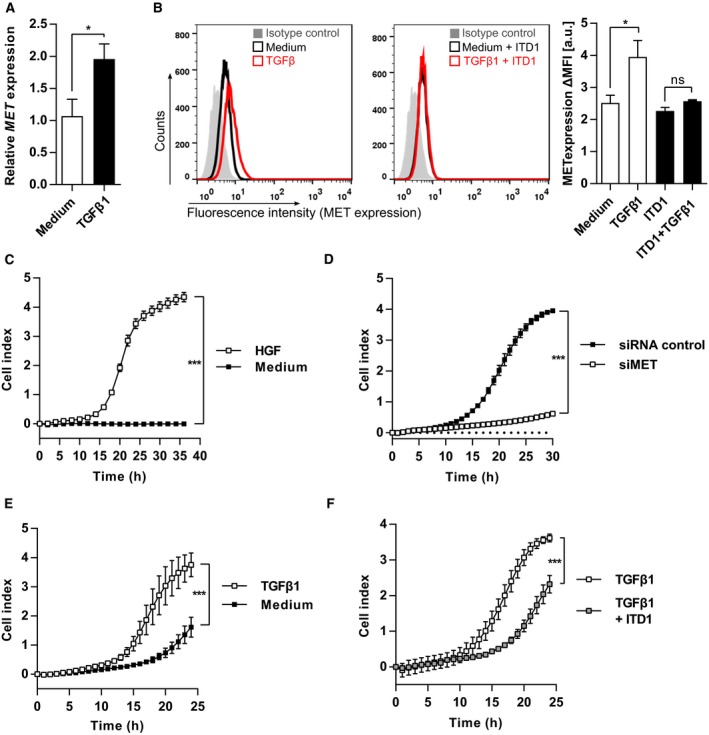
TGFβ1 elevates expression of MET and induces cell migration in MCF10A cell line. (A) Gene expression analysis using quantitative PCR in MCF10A cells, which were treated with 10 ng·mL^−1^ TGFβ1 or untreated (medium) (*n* = 3; Student's unpaired *t*‐test). (B) Flow cytometry analysis of MCF10A cells after TGFβ1 treatment in combination with or without 2.5 μm TGFBR2 inhibitor ITD1 (*n* = 3). (C) Migration analysis of MCF10A cells toward HGF using the RTCA. (*n* = 4). (D) Migration analysis of MCF10A cells toward HGF using the RTCA. MCF10A cells were transfected with a pool of anti‐MET siRNAs or siRNA control prior to the migration assay. Cells were seeded in starvation medium, and HGF was used as chemoattractant (*n* = 3). (E) MCF10A cells were treated with or without TGFβ1 for 72 h prior to the migration assay. Cells were seeded in starvation medium, and HGF was used as chemoattractant (*n* = 4). (F) MCF10A cells were treated with TGFβ1 in combination with or without ITD1 for 72 h prior to the migration assay. Cells were seeded in starvation medium, and HGF was used as chemoattractant (*n* = 3). Data are expressed as mean ± SD. The significance was determined using Student's unpaired *t*‐test. Key: *P < 0.05; ***P < 0.001.

We hypothesized that the observed correlation between TGFBR2 and MET is based on a common regulatory mechanism. To this end, we activated MET and TGFβ signaling pathways with HGF and TGFβ1, respectively, and tested their influence on TGFBR2 or MET expression in the model cell line MCF10A as well as in basal‐like breast cancer cell lines HS578T and HCC1143. TGFβ1 significantly increased gene and surface protein expression of MET (Figs 3A, B and S5), while HGF did not affect *TGFBR2* expression (Fig. S6). Next, we blocked TGFβ signaling with the highly selective TGFBR2 inhibitor ITD1 (Willems *et al*., 2012), which obstructed TGFβ1‐induced MET expression (Fig. 3B) and proved a regulation of MET by the TGFβ signaling pathway.

Besides TGFβ, MET signaling importantly contributes to cell migration and metastasis formation (Birchmeier *et al*., 2003; Ho‐Yen *et al*., 2015). Thus, we hypothesized that HGF could also influence cell migration *in vitro*. Indeed, we could show that HGF induces cell migration (Fig. 3C) and that this phenotype could be blocked by knockdown of MET using single as well as pooled anti‐MET siRNAs (Figs 3D and S7A, B).

Next, we hypothesized that the strong upregulation of MET by TGFβ would also impact on HGF‐mediated migration *in vitro*. To this end, we treated MCF10A, HS578T, and HCC1143 cells with TGFβ1 and could show stronger migration capabilities toward HGF (Figs 3E and S7C), which could be downregulated by the addition of the TGFBR2 inhibitor ITD1 (Figs 3F and S7D).

In conclusion, these results demonstrate that TGFβ1 upregulates MET and thereby leads to increases in migration in an HGF‐dependent manner.

### 3.3 MET expression is regulated via C‐ets‐1

**Figure 4:**
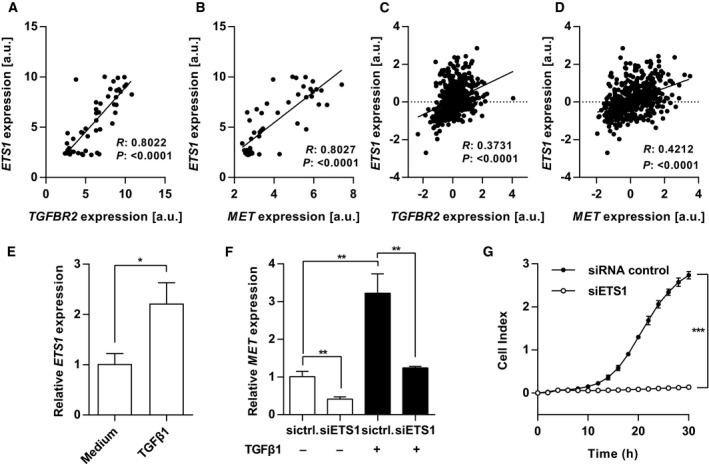
C‐ets‐1 regulates MET expression induced by TGFβ1. (A+B) Correlation analysis of (A) *ETS1* and *TGFBR2* and (B) *ETS1* and *MET* mRNA expression in breast cancer cell lines with each data point representing a different breast cancer cell line [Pearson's correlation (*R*); breast cancer cell line microarray database, *n* = 51 (Riaz *et al*., 2013)]. (C+D) Correlation analysis of (C) *ETS1* and *TGFBR2* and (D) *ETS1* and *MET* mRNA expression in breast cancer patients with each data point representing an individual sample [Pearson's correlation (*R*); TCGA breast cancer dataset, *n* = 547]. (E) Quantitative PCR for *ETS1* mRNA expression. MCF10A cells were treated with 10 ng·mL^−1^ TGFβ1 for 48 h. Gene expression revealed increased *ETS1* expression after TGFβ1 treatment (*n* = 3; Student's unpaired *t*‐test). (F) Quantitative PCR of *MET* mRNA expression. MCF10A cells previously transfected with siRNAs against *ETS1* as well as with the negative control and treated with 10 ng·mL^−1^ TGFβ1 for 48 h (*n* = 3; Student's unpaired *t*‐test). (G) Cell migration of MCF10A cells previously transfected with siRNAs against *ETS1* as well as with the negative control and was assessed by an RTCA trans‐well migration assay. Cells were seeded in starvation medium and allowed to migrate using starvation medium plus 75 ng·mL^−1^ HGF as chemoattractant (*n* = 3; Student's unpaired *t*‐test). Data are expressed as mean ± SD. Key: *P < 0.05; **P < 0.01; ***P < 0.001.

Next, we wanted to identify the mechanism of TGFβ1‐induced expression of MET. To this end, we bioinformatically searched for putative transcription factor binding sites in the MET promoter and combined this with an analysis of genes that are coexpressed with either *TGFBR2* or *MET* in breast cancer cell lines. Several transcription factors were found to have predicted binding sites in the MET promoter (Table S4). C‐ets‐1 was the top candidate as this transcription factor has previously been described to synergize with SMAD3 (Lindemann *et al*., 2001) and its expression also correlated the most with expression of *TGFBR2* as well as of *MET* in breast cancer cell lines (Fig. 4A, B, Tables S3 and S5). This strong correlation was validated in an independent dataset of breast cancer cell lines (Fig. S8A, B) (Kao *et al*., 2009). Correlation of *ETS1* with *MET* and *TGFBR2* was observed as well in breast cancer tissue using the TCGA breast cancer dataset (Fig. 4C, D).

We thus hypothesized that C‐ets‐1 is regulated by TGFβ1. To test this, we stimulated MCF10A, HS578T, and HCC1143 cells with TGFβ1 and then measured the expression levels of *ETS1*. We observed increases in *ETS1* expression (Figs 4E and S9A), supporting a potential role of C‐ets‐1 as regulator of TGFβ1‐induced MET expression. To ascertain this regulation of C‐ets‐1 by TGFβ1, we knocked down C‐ets‐1 leading to decreased basal MET expression at the RNA level in MCF10A, HS578T, and HCC143 cells (Figs 4F and S9B–D) and resulted in a reduction of HGF‐induced MET phosphorylation (Fig. S9E). C‐ets‐1 knockdown also decreased TGFβ1‐induced MET expression (Figs 4F and S9F). These results suggest that C‐ets‐1 is involved in HGF‐mediated cell migration. Accordingly, we knocked down C‐ets‐1 in MCF10A as well as HCC1143 cells and then analyzed HGF‐dependent cell migration. As expected, knock down of C‐ets‐1 completely blocked or reduced cell migration toward HGF in MCF10A and HCC1143 cell, respectively (Figs 4G and S9G). These results show that TGFβ1 regulates MET expression at least in part via upregulation of C‐ets‐1 and defines both molecules as critical factors for HGF‐induced cell migration.

### 3.4 MiR‐128‐3p negatively correlates with MET expression in breast cancer and inhibits HGF‐induced cell migration by directly targeting *MET*

**Figure 5:**
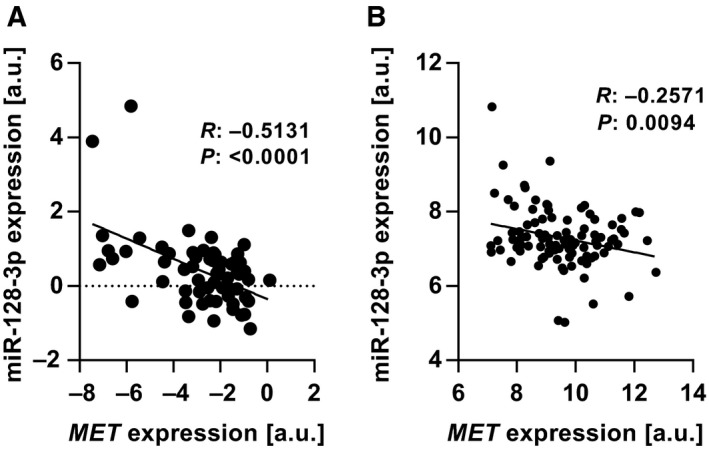
miR‐128‐3p negatively correlates with *MET* expression. (A) Correlation analysis of miR‐128‐3p miRNA and *MET* mRNA expression in the NCI‐60 cell line panel each data point representing a different cancer cell line [Pearson's correlation (*R*); *n* = 60]. (B) Correlation analysis of miR‐128‐3p miRNA and *MET* mRNA expression in breast cancer patient dataset with each data point representing an individual sample [Pearson's correlation (*R*); METABRIC,* n* = 779].

We next wanted to know whether also miRNAs would potentially contribute to coordinated MET regulation via TGFβ1 to better reflect the complexity of MET transcriptional and post‐transcriptional regulation. To this end, we first screened the TargetScan database for miRNAs that could regulate MET expression. miR‐128‐3p was among the top predicted candidates, and its expression was found to negatively correlate with *MET* in a cell line panel (Fig. 5A). The same was observed upon analysis of the METABRIC breast tumor tissue dataset (Fig. 5B), which also showed lower expression of miR‐128‐3p in more aggressive ER‐negative and basal‐like breast cancer compared to ER‐positive and luminal A and B breast cancer (Fig. S10A, B).

**Figure 6:**
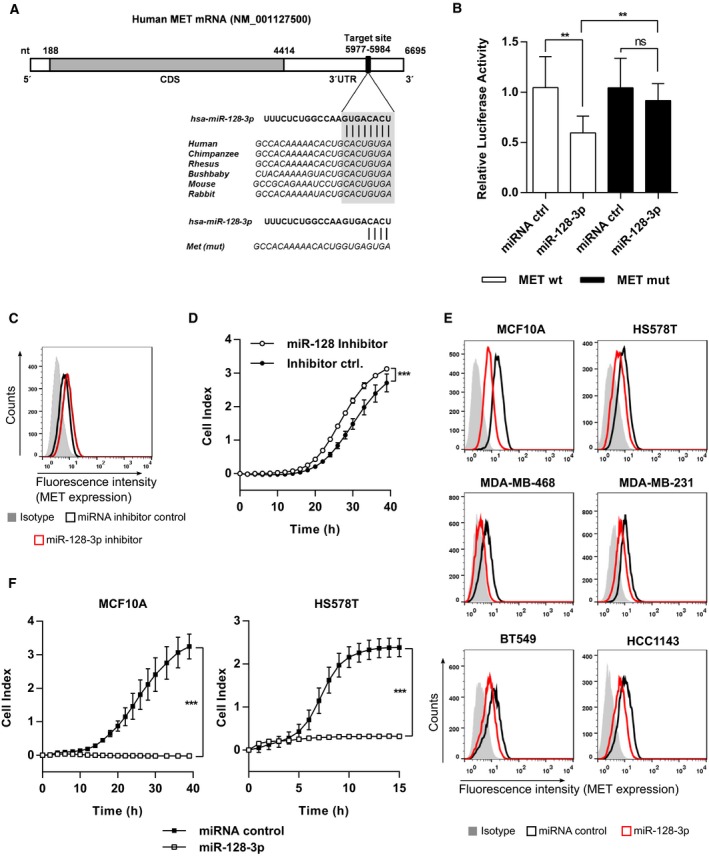
miR‐128‐3p directly targets MET expression and inhibits HGF‐induced cell migration. (A) Schematic representation of the *MET* mRNA with the predicted target sites for miR‐128‐3p in the 3′UTR. This region is conserved among different species. (B) Luciferase reporter assay in MCF‐7 cells transfected with vectors containing either wild‐type (wt) or mutated (mut) 3′ UTRs of *MET* and miR‐128‐3p mimic or negative control (*n* = 6; Student's unpaired *t*‐test). (C) MET protein levels after transfection of MCF10A cell line with miR‐128‐3p inhibitor or miRNA inhibitor control. Surface molecules expression was analyzed 48 h after transfection with flow cytometry. (D) Cell migration of MCF10A cells previously transfected with antisense‐miR‐128‐3p and negative control was assessed by RTCA trans‐well migration assay. Cells were seeded in starvation medium and allowed to migrate using starvation medium plus 75 ng·mL^−1^ HGF as chemoattractant (*n* = 4; Student's unpaired *t*‐test). (E) MET protein levels after transfection of basal‐like breast cancer and epithelial cells with miR‐128‐3p mimics or mimic control. Surface molecules expression was analyzed 48 h after transfection with flow cytometry. (F) Cell migration of MCF10A and HS578T cells previously transfected with miR‐128‐3p mimics and mimic control was assessed by RTCA migration assay. HGF was used as chemoattractant (*n* = 4; unpaired *t*‐test). Data are expressed as mean ± SD; Key: *P < 0.05; **P < 0.01; ***P < 0.001.

We thus hypothesized that miR‐128‐3p directly targets MET, as there is one highly conserved and predicted binding site for miR‐128‐3p in the 3′UTR of the MET mRNA (Fig. 6A). The 3′UTR of *MET* was cloned into a luciferase reporter system, and luciferase‐based reporter assays were performed with wild‐type or mutated binding sites for miR‐128‐3p. Luciferase activity of the MET wild‐type 3′UTR construct was significantly decreased when miR‐128‐3p was cotransfected. This effect could be rescued by cotransfection of the plasmid containing the mutated 3′UTR, which proved direct targeting of miR‐128‐3p at the predicted binding site within the *MET* 3′UTR (Fig. 6B). It has been reported that TGFβ1 downregulates miR‐128‐3p expression in breast cancer cell lines (Qian *et al*., 2012), which could be confirmed in our model system MCF10A (Fig. S11A). We thus hypothesized that TGFβ1‐mediated downregulation of miR‐128‐3p would increase *MET* expression. To this end, we tested whether antagomirs against miR‐128‐3p (Fig. S11B) could mimic the effect of TGFβ1 on increasing *MET* expression. Indeed, inhibition of miR‐128‐3p significantly induced *MET* expression in MCF10A, HS578T, and HCC1143 cells (Figs 6C and S11C). Based on these results, we hypothesized that miR‐128‐3p inhibition could also modulate HGF‐induced cell migration in MCF10A cells. Therefore, the cells were transfected with miR‐128‐3p antagomirs and HGF‐induced migration was measured 48 h later. miR‐128‐3p antagomirs increased migration of MCF10A cells toward HGF and confirmed the activity of miR‐128‐3p in this process (Fig. 6D). Next, we tested the impact of miR‐128‐3p on MET expression in MET‐positive breast cancer cell lines. As expected, surface protein levels of MET were reduced in MCF10A as well as in all tested basal‐like breast cancer cell lines HS578T, MDA‐MB‐468, MDA‐MB‐231, BT549, and HCC1143 upon overexpression of miR‐128‐3p (Fig. 6E). Ectopic overexpression of miR‐128‐3p using respective mimics (Fig. S11D) strongly reduced HGF‐mediated migration in MCF10A as well as in HS578T cell lines (Fig. 6F).

Altogether, these data demonstrate that TGFβ1 modulates the expression levels of C‐ets‐1 and miR‐128‐3p, thereby triggering promigratory stimuli from the tumor microenvironment in breast epithelial and cancer cells.

## 4 Discussion

Breast cancer is a highly heterogeneous disease and the clinical outcome strongly correlates with the respective tumor subtype (Jemal *et al*., 2011). The high mortality rate in patients diagnosed with specific subtypes of breast cancer derives from the limited therapeutic success and a rapid onset of metastasis. Tumor cells and cells of the tumor microenvironment secrete diverse growth factors and cytokines and establish a specific milieu fostering tumor growth, tumor cell migration, and angiogenesis (Quail and Joyce, 2013). In this context, TGFβ1 and MET form key players and act on both cancer cells and the tumor stroma. In breast cancer, TGFβ1 is upregulated compared to its adjacent nonmalignant tissue and it impacts on various cells of the tumor microenvironment. In the tumor stroma, TGFβ1 gets secreted by tumor‐associated macrophages and CAFs (Kojima *et al*., 2010; Yu *et al*., 2014). It has been shown that TGFβ1 directly acts on the basal‐like breast tumor cell line MDA‐MB‐231 and that this drives metastatic processes of breast cancer cells by its binding to TGFBR2 (Willis *et al*., 2015). In line with these findings, we demonstrate that TGFBR2 is expressed at higher levels in more aggressive basal‐like breast cancer cell lines as well as in triple‐negative breast cancer tissue than in luminal breast cancer cell lines and luminal‐like tumor tissue. This subtype‐specific expression of TGFBR2 might explain why TGFBR2 expression correlates with reduced overall survival of patients with ER‐negative but not with ER‐positive breast cancer (Buck *et al*., 2004). Nevertheless, the exact mechanisms of TGFβ1/TGFBR2‐driven breast cancer progression and metastasis have not been fully elucidated until now.

Here, we highlight the role of TGFβ1 as inducer of tumor cell migration by increasing HGF/MET signaling *in vitro*. We could establish a positive correlation of TGFBR2 and MET protein expression in breast cancer by analyzing 801 tumor tissues samples. In line with our findings for TGFBR2, we observed higher MET expression in nonluminal compared to luminal breast cancer cell lines as well as nonluminal‐like compared to luminal‐like breast tumors. These findings are supported by Xu *et al*. (2012) who identified elevated MET signaling and caveolin accumulation in human basal‐like breast tumors. In addition, high MET expression has been shown to be associated with poor patient survival in breast cancer (Ponzo *et al*., 2009; Raghav *et al*., 2012). MET signaling initiates tumorigenesis in early progenitor cells and induces an invasive growth phenotype, which can be explained by its capacity to increase tumor cell motility, invasion, and resistance toward apoptosis induction (Comoglio and Trusolino, 2002; Graveel *et al*., 2009). In renal epithelial cells, Sp1 and Smad are involved in TGFβ1‐induced MET expression (Zhang *et al*., 2005). However, little is known about how MET expression is regulated downstream of TGFβ signaling in breast cancer.

**Figure 7:**
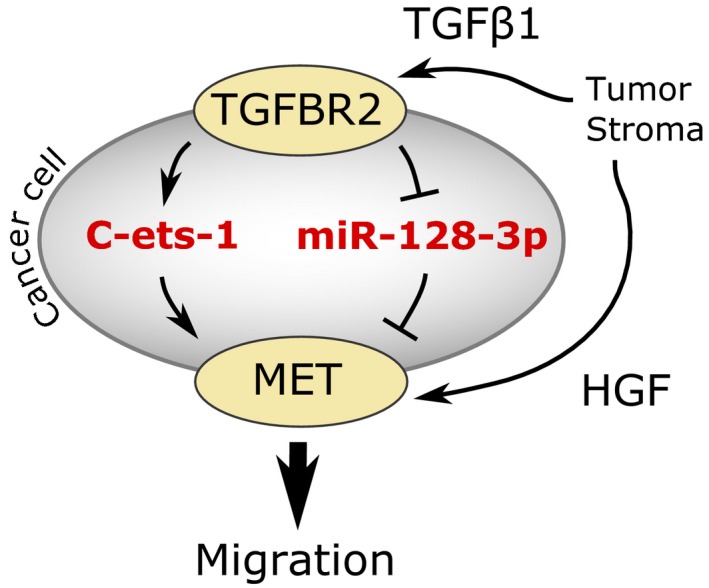
Schematic representation TGFβ1 as a regulator of HGF‐induced cell migration in breast cancer. TGFβ1 induces migration by both increasing C‐ets‐1 levels and by decreasing expression of miR‐128‐3p, which results in elevated levels of MET.

We are the first to show that two different mechanisms of MET regulation are merged by TGFβ1 in aggressive breast cancer cell lines and tumor tissue. The first mechanism describes TGFβ1 as regulator of the transcription factor C‐ets‐1, which mediates elevated expression of *MET*. This finding is supported by Gambarotta *et al*. (1996) as well as by Kubic *et al*., (2015), who both described transcriptional regulation of MET by C‐ets‐1, however, not in a TGFβ1‐dependent context. C‐ets‐1 overexpression has been shown to strongly promote malignant invasiveness of breast cancer cells, which is in line with our findings (Furlan *et al*., 2008). Further, it is prognostic marker for poor prognosis of human breast cancer (Span *et al*., 2002). The second mechanism of MET regulation by TGFβ1 is related to suppression of miR‐128‐3p which, in turn, directly targets *MET*, which is in line with Jiang and colleagues non‐small cell lung cancer (Jiang *et al*., 2016), and thereby impairs HGF/MET‐mediated cell migration. Migliore and colleagues support the idea of post‐transcriptional regulation of MET by showing that also miR‐34b, miR‐34c, and miR‐199a‐5p negatively regulate MET expression and thereby inhibit invasive growth in different cancer cell lines (Migliore *et al*., 2008). In bovine skeletal muscle satellite cells, miR‐128‐3p directly target SP1 through this inhibiting cell proliferation (Dai *et al*., 2016). As SP1 is involved in TGFβ1‐induced MET expression (Zhang *et al*., 2005) in renal epithelial cells, MET might be also regulated by miR‐128‐3p via SP1 in breast cancer. The regulatory mechanism of miR‐128‐3p seems to be relevant also in breast cancer patients as our analysis suggests that miR‐128‐3p is lower expressed in nonluminal breast cancer compared to luminal‐like subtypes. Low‐level expression of miR‐128‐3p also correlates with poor clinical outcome of patients with breast cancer (Qian *et al*., 2012; Zhu *et al*., 2011). Other studies have shown that miR‐128‐3p is also lower expressed in other tumor tissues and that its overexpression inhibits tumor cell proliferation, angiogenesis, and invasion (Evangelisti *et al*., 2009; Hu *et al*., 2014; Palumbo *et al*., 2013). Hence, we define TGFβ1 as a regulator of MET expression in breast cancer by upregulating C‐ets‐1 and downregulating miR‐128‐3p expression, which results in cell migration, invasion, and metastasis in a HGF‐dependent manner (Fig. 7). Regulation of MET expression by TGFβ1 seems to be tumor‐type‐specific as in glioblastoma, TGFβ suppresses HGF/MET pathway activity (Papa *et al*., 2017).

Recent data suggest that miR‐128‐3p is involved in a negative feedback loop of TGFβ signaling in non‐small cell lung cancer (Cai *et al*., 2017). While we have shown that TGFβ1 results in downregulation of miR‐128‐3p, Cai and colleagues demonstrated that the negative regulators of TGFβ signaling, SMURF2 and PP1c, are regulated by miR‐128‐3p (Cai *et al*., 2017). However, aggressive breast cancer carries elevated TGFβ signaling activities, which indicates that this negative feedback loop is not effective in this cancer entity (Dunning *et al*., 2003). Further, expressions of SMURF2 as well as of PP1c neither correlated with miR‐128‐3p expression nor were these genes differentially expressed in the different breast cancer subtypes (data not shown). Hence, the regulation of events that are controlled by TGFβ signaling seems to be intricately controlled and this regulation appears to be context‐dependent.

Currently, several TGFβ and MET pathway inhibitors are under development. A few have already been tested in clinical trials and show promising results in various cancer entities, including metastatic breast cancer (Ganapathy *et al*., 2010; Gherardi *et al*., 2012). By revealing TGFβ1 as a regulator of HGF/MET‐induced cell migration, our findings suggest TGFβ1 with its downstream mediators C‐ets‐1 and miR‐128‐3p as potential targets for therapy of basal‐like breast cancer.

## 5 Conclusions

In this study, we demonstrate that TGFβ signaling leads to increased MET signaling activity and that this results in HGF‐dependent migration of breast epithelial and cancer cells. We could explain this migratory phenotype by the action of TGFβ1 leading to both upregulation of C‐ets‐1 and inhibition of miR‐128‐3p expression. Higher C‐ets‐1 and lower miR‐128‐3p expression levels intensify MET signaling and thereby suggest TGFβ1 as a regulator of HGF‐mediated migration in MCF10A and breast cancer cells.

## Author contributions

CB designed the study. CB, NE, AB, JG, SB, CG, and ER performed experiments. AW, TB, and SB analyzed RPPA data of patient samples. EK and MV carried out material support and preparation. CB, NE, JG, AB, TB, and SW interpreted the results, and CB, AB, and SW wrote the manuscript. All authors have reviewed and approved the manuscript for submission.

## Supporting information


**Fig. S1.** Specificity of MET and TGFBR2 antibodies.
**Fig. S2.** TGFBR2 is higher expressed in estrogen receptor negative breast cancer cell lines.
**Fig. S3.** *MET* correlates with *TGFBR2* expression.
**Fig. S4.** *MET* is higher expressed in estrogen receptor negative breast cancer cell lines.
**Fig. S5.** TGFβ1 elevates expression of MET in HCC1143 and HS578T cell lines.
**Fig. S6.** HGF does not induce *TGFBR2* expression.
**Fig. S7.** Cell migration is regulated by MET and TGFβ1.
**Fig. S8.** *ETS‐1* correlates with *TGFBR2* and *MET* expression.
**Fig. S9.** Interference of MET by C‐ets‐1.
**Fig. S10.** miR‐128‐3p is lower expressed in basal‐like breast cancer.
**Fig. S11.** Regulation of miR‐128‐3p and *MET* expression by TGFβ1 and miR‐128‐3p.
**Table S1.** Sequences of siRNAs targeting *MET*,* TGFBR2*,* ETS1* as well as non‐targeting siRNA control.
**Table S2.** Sequences and Universal Probe Library (UPL) probe numbers for genes quantified by TaqMan qRT‐PCR.
**Table S3.** Correlation analysis of *TGFBR2* in a data set (Riaz *et al*.) containing mRNA expression of 51 breast cancer cells. Shown are the top 33 correlated genes.
**Table S4.** Transcription factors correlated with (A) *TGFBR2* and (B) *MET* having putative binding site in the MET promoter.
**Table S5.** Correlation analysis of *MET* in a data set (Riaz *et al*.) containing mRNA expression of 51 breast cancer cells. Shown are the top 33 correlated genes.
