# Supplementary material for: TGFβ1 regulates HGF‐induced cell migration and hepatocyte growth factor receptor MET expression via C‐ets‐1 and miR‐128‐3p in basal‐like breast cancer
Source: Mol Oncol. 2018 Jul 30;12(9):1447–63. doi: 10.1002/1878-0261.12355 (PMC6120235; doi:10.1002/1878-0261.12355)
Supplement: Supplementary file 1 — Fig. S1. Specificity of MET and TGFBR2 antibodies. Fig. S2. TGFBR2 is higher expressed in estrogen receptor negative breast cancer cell lines. Fig. S3. MET correlates with TGFBR2 expression. Fig. S4. MET is higher expressed in estrogen receptor negative breast cancer cell lines. Fig. S5. TGFβ1 elevates expression of MET in HCC1143 and HS578T cell lines. Fig. S6. HGF does not induce TGFBR2 expression. Fig. S7. Cell migration is regulated by MET and TGFβ1. Fig. S8. ETS‐1 correlates with TGFBR2 and MET expression. Fig. S9. Interference of MET by C‐ets‐1. Fig. S10. miR‐128‐3p is lower expressed in basal‐like breast cancer. Fig. S11. Regulation of miR‐128‐3p and MET expression by TGFβ1 and miR‐128‐3p. Table S1. Sequences of siRNAs targeting MET, TGFBR2, ETS1 as well as non‐targeting siRNA control. Table S2. Sequences and Universal Probe Library (UPL) probe numbers for genes quantified by TaqMan qRT‐PCR. Table S3. Correlation analysis of TGFBR2 in a data set (Riaz et al.) containing mRNA expression of 51 breast cancer cells. Shown are the top 33 correlated genes. Table S4. Transcription factors correlated with (A) TGFBR2 and (B) MET having putative binding site in the MET promoter. Table S5. Correlation analysis of MET in a data set (Riaz et al.) containing mRNA expression of 51 breast cancer cells. Shown are the top 33 correlated genes. [file MOL2-12-1447-s001.pdf]

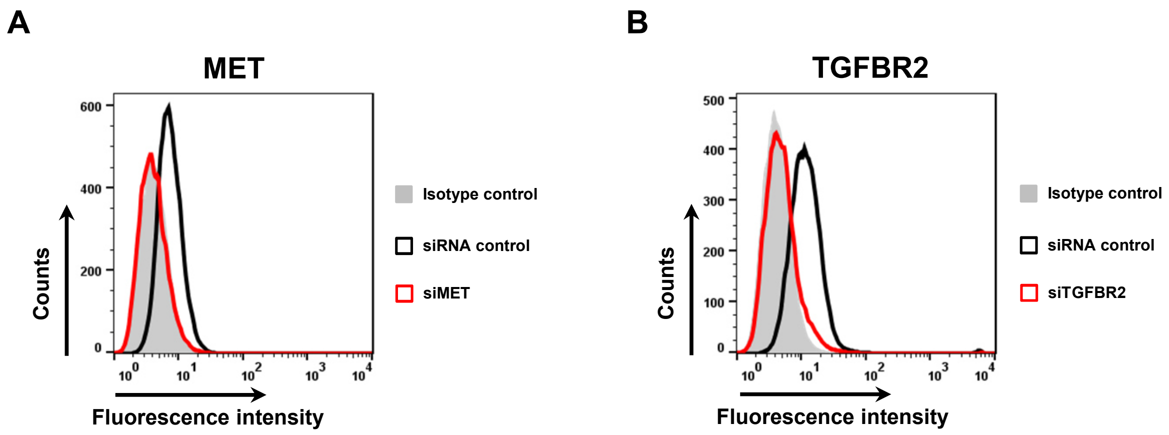

Fig. S1. Specificity of MET and TGFBR2 antibodies. Flow cytometry analysis of (A) MET and (B) TGFBR2 cell surface molecule expression on MCF10A cell line using siRNA against (A) MET and (B) TGFBR2, respectively. As a control a non-targeting siRNA was used.

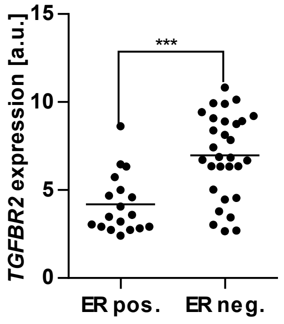

Fig. S2. *TGFBR2* is higher expressed in estrogen receptor negative breast cancer cell lines. Quantitative PCR for *TGFBR2* mRNA expression in breast cancer cell lines using breast cancer cell line microarray database for *TGFBR2* (Riaz et al 2013). Analysis revealed higher *TGFBR2* expression in estrogen receptor negative (ER neg.) than estrogen receptor positive (ER pos.) breast cancer cell lines (ER pos. n=18; ER neg. n=30; Student's unpaired t-test). Mean is indicated as line. Key: ***$p < 0.001$.

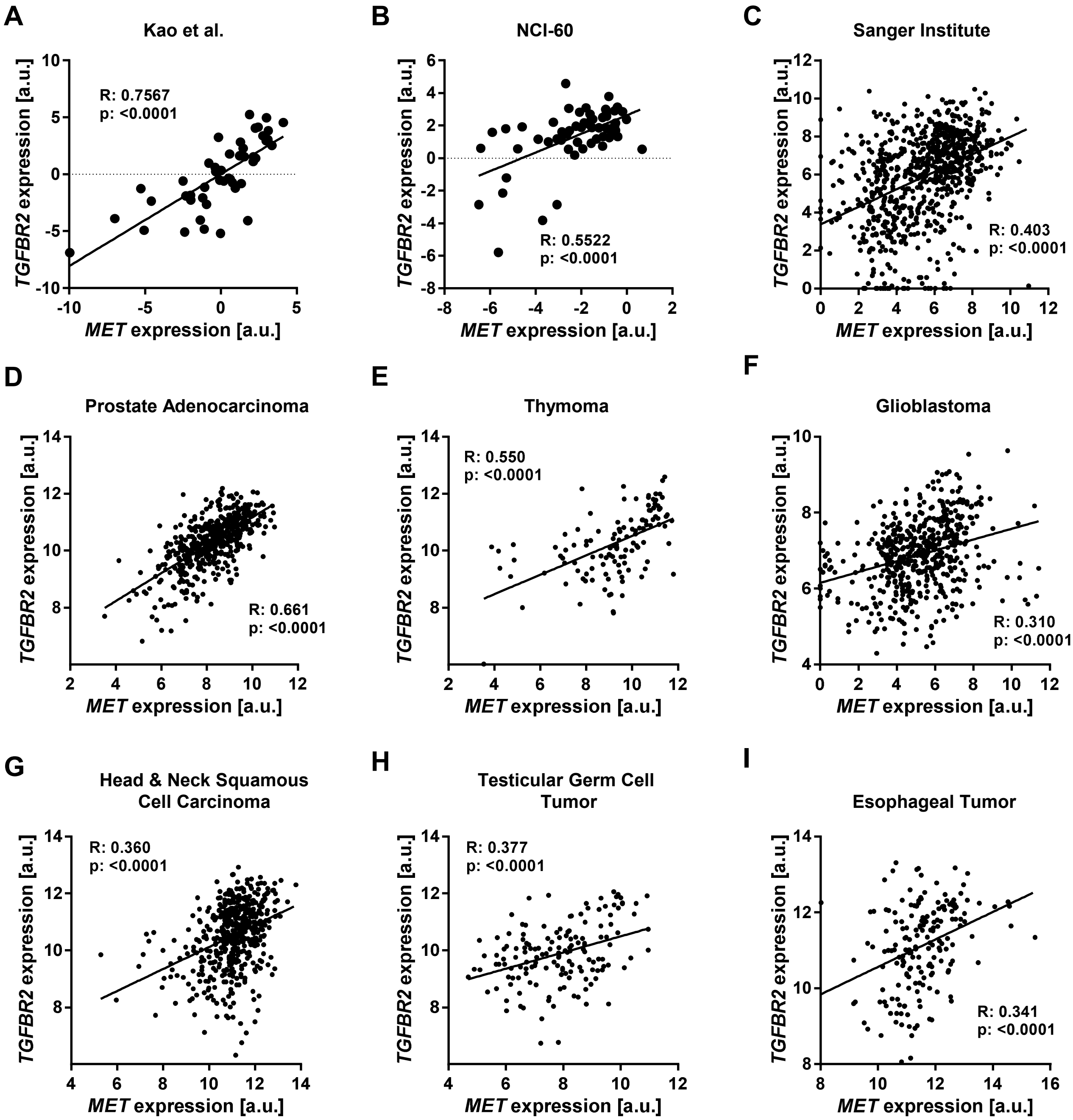
A
Kao et al.
R: 0.7567
p: <0.0001
TGFBR2 expression [a.u.]
MET expression [a.u.]
B
NCI-60
R: 0.5522
p: <0.0001
C
Sanger Institute
R: 0.403
p: <0.0001
D
Prostate Adenocarcinoma
R: 0.661
p: <0.0001
E
Thymoma
R: 0.550
p: <0.0001
F
Glioblastoma
R: 0.310
p: <0.0001
G
Head & Neck Squamous Cell Carcinoma
R: 0.360
p: <0.0001
H
Testicular Germ Cell Tumor
R: 0.377
p: <0.0001
I
Esophageal Tumor
R: 0.341
p: <0.0001

Fig. S3. *MET* correlates with *TGFBR2* expression. (A-C) Correlation analysis of *MET* and *TGFBR2* mRNA expression in (A) breast cancer cell lines with each data point representing a different breast cancer cell line, (B) different cancer cell lines of the NCI-60 cell line panel with each data point representing a different cell line and (C) different cancer cell lines of the Sanger cell line panel with each data point representing a different cell line and correlation coefficient (R) indicated (Pearson correlation; breast cancer cell line microarray database, n=48 (Kao et al., 2009); NCI-60 cell line panel, n=60; Sanger cell line panel, n=789). (D-I) Correlation analysis of MET and TGFBR2 mRNA expression in (D) prostate adenocarcinoma patients, (E) thymoma patients, (F) glioblastoma patients, (G) head and neck squamous cell carcinoma patients, (H) testicular germ cell tumor patients and (I) esophageal tumor patients with each data point representing an individual sample and correlation coefficient (R) indicated (Pearson correlation; (D) TCGA prostate adenocarcinoma data set, n=497; (E) TCGA thymoma data set, n=120; (F) TCGA glioblastoma data set, n=540; (G) TCGA head and neck squamous cell carcinoma data set, n=520; (H) TCGA testicular germ cell tumor data set, n=150; (I) TCGA esophageal tumor data set, n=184).

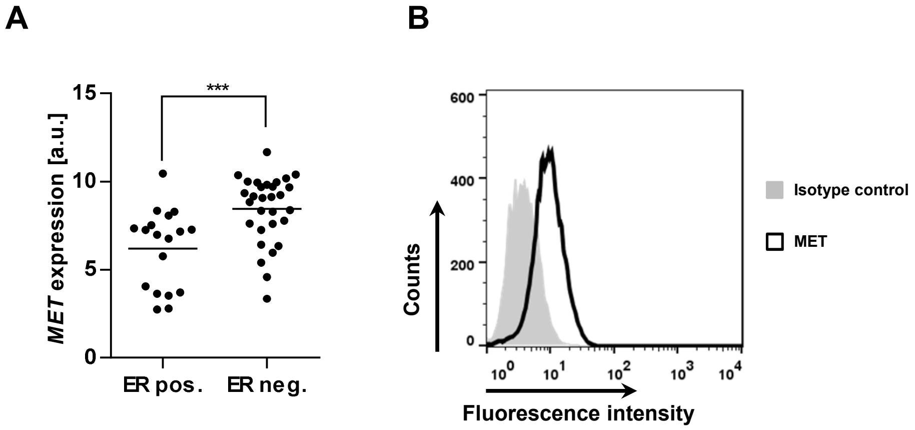

Fig. S4. ***MET* is higher expressed in estrogen receptor negative breast cancer cell lines.** (A) Quantitative PCR for *MET* mRNA expression in breast cancer cell lines using breast cancer cell line microarray database for *MET* (Riaz et al 2013). Analysis revealed higher *MET* expression in estrogen receptor negative (ER neg.) than estrogen receptor positive (ER pos.) breast cancer cell lines (ER pos. n=18; ER neg. n=30; Student's unpaired t-test). (B) Flow cytometry analysis of basal HER2+ breast cancer cell line HCC1954 for c-Met cell surface molecule expression. Data are expressed as mean. Key: ***p < 0.001

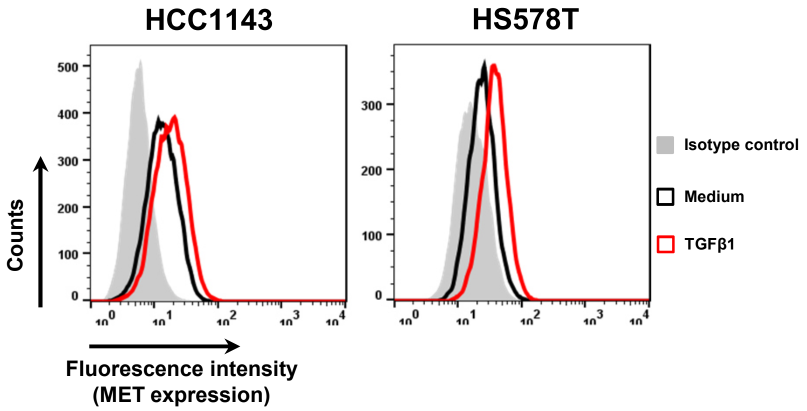

Fig. S5. TGFβ1 elevates expression of MET in HCC1143 and HS578T cell lines. Flow cytometry analysis of MET cell surface molecule expression on HCC1143 and HS578T cells after TGFβ1 treatment for 72 hours.

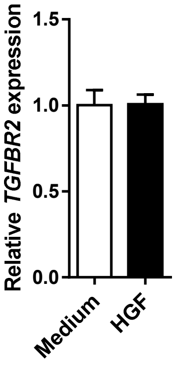

Fig. S6. HGF does not induce *TGFBR2* expression. Quantitative PCR for *TGFBR2* mRNA expression in MCF10A cells treated for 48 hours with 75 ng/ml HGF. Data are expressed as mean + SD.

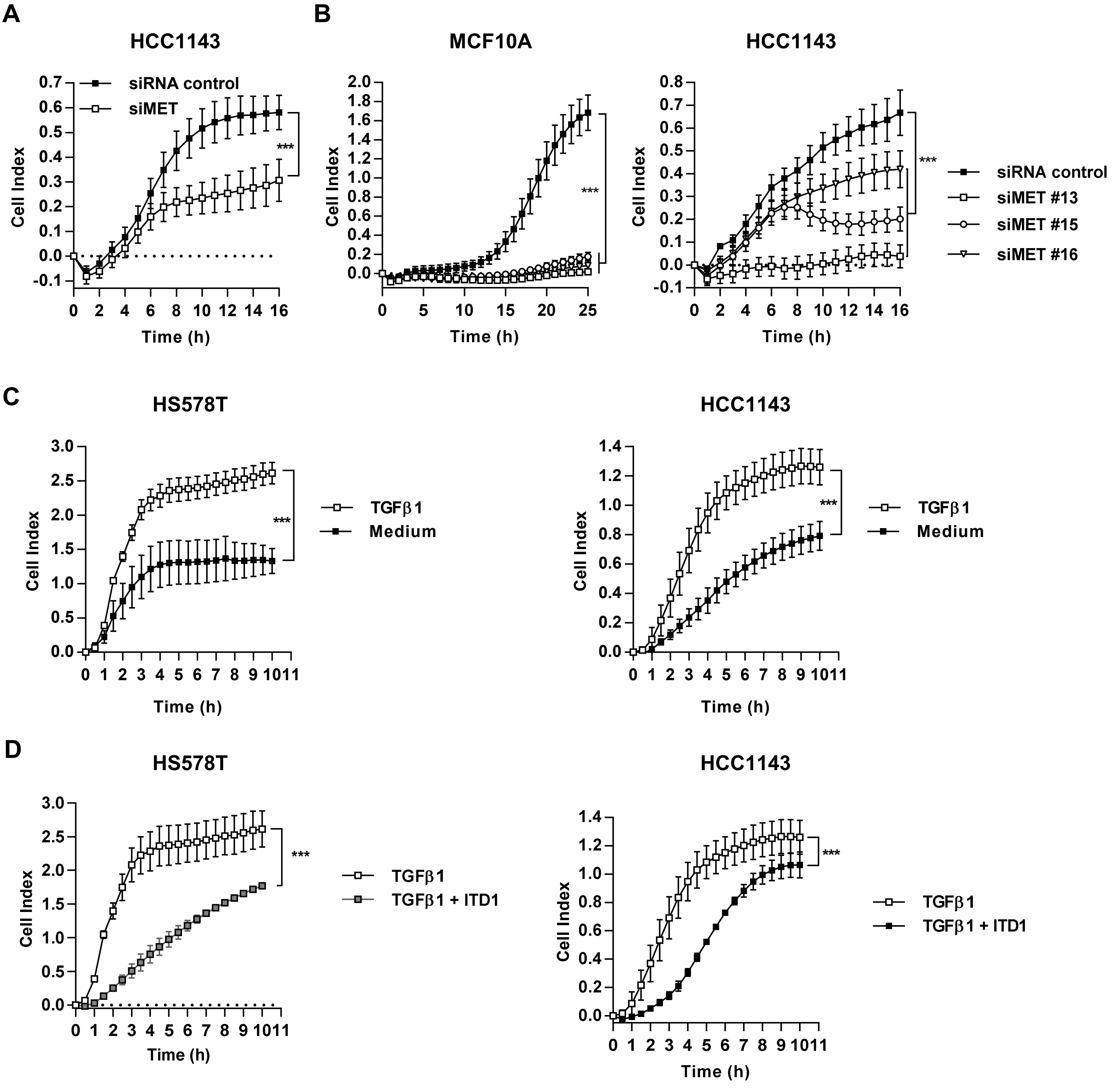

A
HCC1143
siRNA control
siMET
Cell Index
Time (h)
***
B
MCF10A
Cell Index
Time (h)
***
HCC1143
Cell Index
Time (h)
***
siRNA control
siMET #13
siMET #15
siMET #16
C
HS578T
Cell Index
Time (h)
***
TGFβ1
Medium
HCC1143
Cell Index
Time (h)
***
TGFβ1
Medium
D
HS578T
Cell Index
Time (h)
***
TGFβ1
TGFβ1 + ITD1
HCC1143
Cell Index
Time (h)
***
TGFβ1
TGFβ1 + ITD1

Fig. S7. Cell migration is regulated by MET and TGFβ1. (A) HCC1143 cells were transfected with a pool of anti-MET siRNAs or siRNA control prior to the migration assay. Cells were seeded in starvation medium and HGF was used as chemoattractant (n=4). (B) MCF10A and HCC1143 cells were transfected with single anti-MET siRNAs or siRNA control prior to the migration assay. Cells were seeded in starvation medium and HGF was used as chemoattractant (n=3). (C) HCC1143 and HS578T cell lines were treated with or without TGFβ1 for 72 hours prior to the migration assay. Cells were seeded in starvation medium and HGF was used as chemoattractant (n=3). (D) HCC1143 and HS578T cell lines were treated with TGFβ1 in combination with or without ITD1 for 72 hours prior to the migration assay. Cells were seeded in starvation medium and HGF was used as chemoattractant (n=3). Data are expressed as mean ± SD. The significance was determined using Student's unpaired t-test. Key: ***$p < 0.001$.

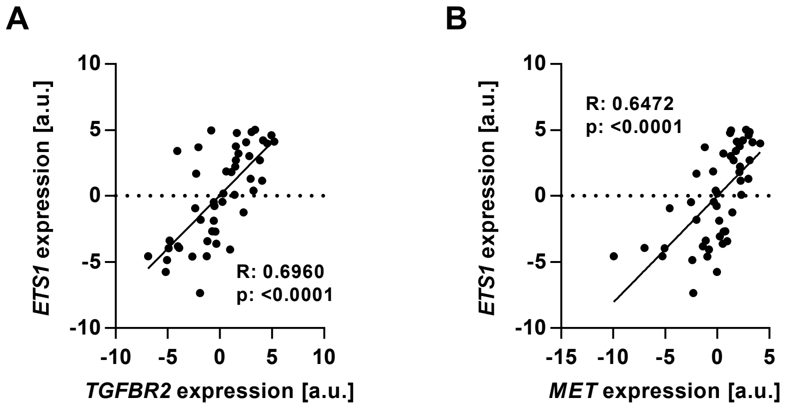

Fig. S8. *ETS-1* correlates with *TGFBR2* and *MET* expression. (A) Correlation analysis of *ETS1* and *TGFBR2* mRNA expression in breast cancer cell lines with each data point representing a different breast cancer cell line and correlation coefficient (R) indicated (Pearson correlation; breast cancer cell line microarray database, n=47 (Kao et al., 2009). (B) Correlation analysis of *ETS1* and *MET* mRNA expression in breast cancer cell lines with each data point representing a different breast cancer cell line and correlation coefficient (R) indicated (Pearson correlation; breast cancer cell line microarray database, n=48 (Kao et al., 2009).

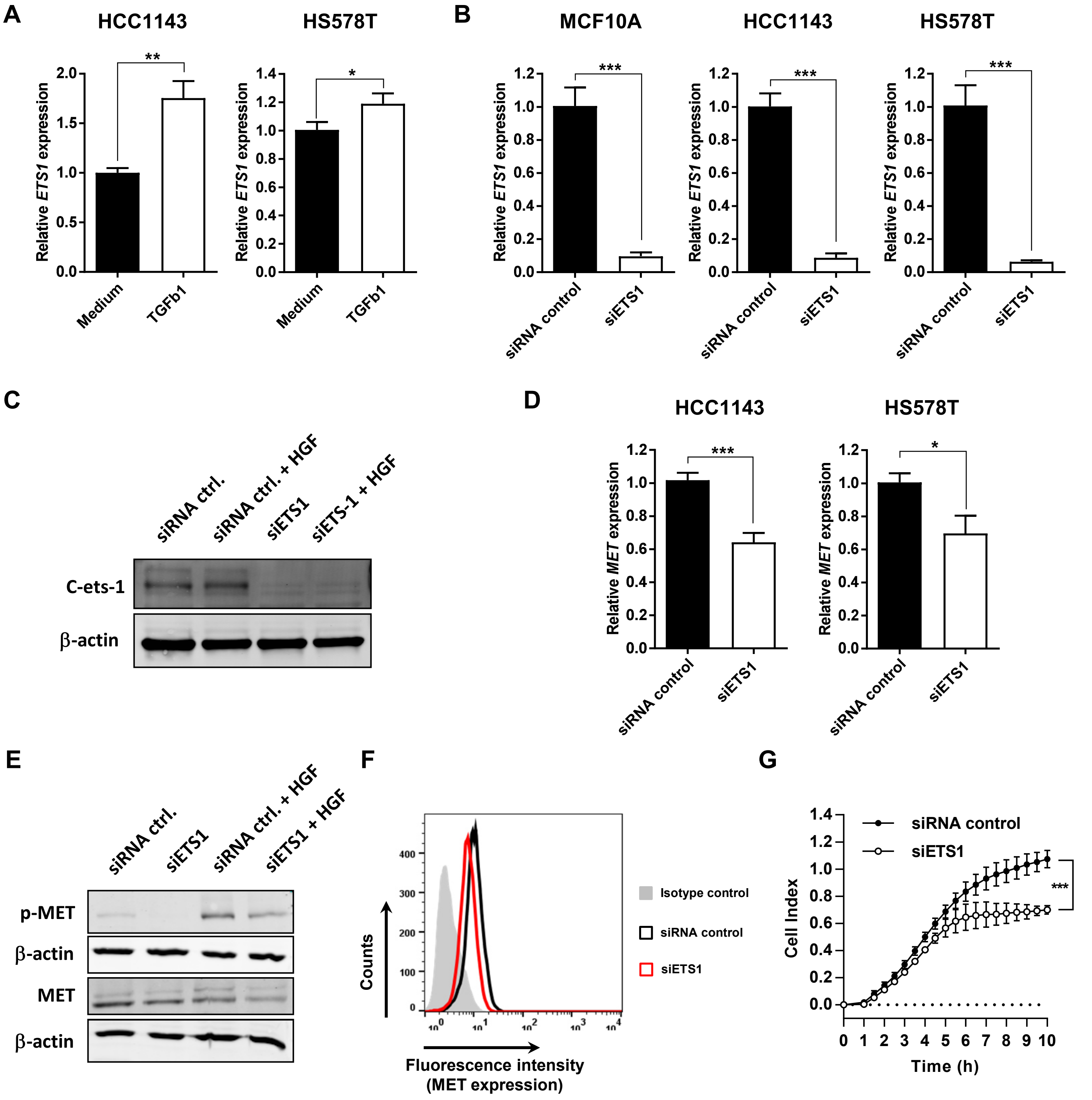

A
HCC1143
HS578T
Relative ETS1 expression
Medium
TGFb1
B
MCF10A
HCC1143
HS578T
siRNA control
siETS1
C
siRNA ctrl.
siRNA ctrl. + HGF
siETS1
siETS-1 + HGF
C-ets-1
β-actin
D
HCC1143
HS578T
Relative MET expression
siRNA control
siETS1
E
siRNA ctrl.
siETS1
siRNA ctrl. + HGF
siETS1 + HGF
p-MET
β-actin
MET
β-actin
F
Counts
Fluorescence intensity
(MET expression)
Isotype control
siRNA control
siETS1
G
siRNA control
siETS1
Cell Index
Time (h)

Fig. S9. Interference of MET by C-ets-1. (A) Quantitative PCR for *ETS1* mRNA expression. HCC1143 and HS578T cells were treated with 10 ng/ml TGFβ1 for 48 hours. Gene expression revealed increased *ETS1* expression after TGFβ1 treatment (n=3; Student's unpaired t-test). (B) Quantitative PCR for *ETS1* mRNA expression in MCF10A, HCC1143 and HS578T cells transfected with siRNA control or siETS1 for 48 hours (n=3; Student's unpaired t-test). (C) Western blot analysis of C-ets-1 expression of MCF10A cells transfected with siRNA control or siETS1 for 48 hours and stimulated with and without 75 ng/ml HGF. (D) Quantitative PCR for *MET* mRNA expression in HCC1143 and HS578T cells transfected with siRNA control or siETS1 for 48 hours (n=3; Student's unpaired t-test). (E) Western blot analysis of phosphorylated and total c-Met expression of MCF10A cells transfected with siRNA control or siETS1 for 48 hours and stimulated with and without 75ng/ml HGF. (F) Flow cytometry analysis of MET cell surface molecule expression on MCF10A cells transfected with siRNA control or siETS1 for 48 hours. (G) Cell migration of HCC1143 cells previously transfected with siRNAs against *ETS1* as well as with the negative control and was assessed by an RTCA trans-well migration assay. Cells were seeded in starvation medium and allowed to migrate using starvation medium plus 75 ng/ml HGF as chemoattractant (n=3; Student's unpaired t-test). Data are expressed as mean ± SD. Key: *$p < 0.05$; **$p < 0.01$; ***$p < 0.001$.

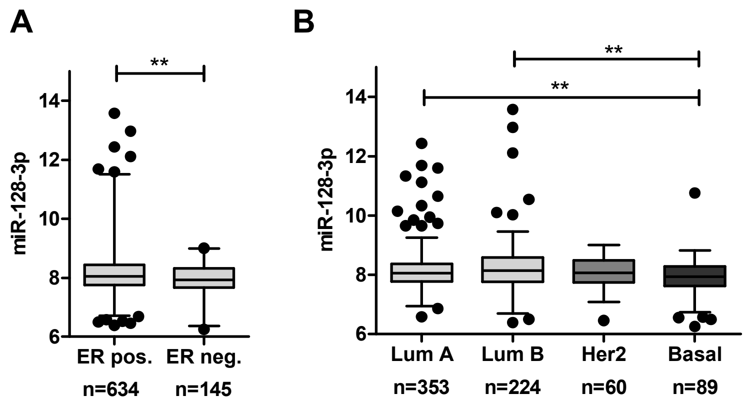

Fig. S10. miR-128-3p is lower expressed in basal-like breast cancer. (A) miR-128-3p expression in breast cancer patients subdivided in an ER negative (n=145) and ER positive (n=634) group of METABRIC expression data set. Horizontal line represents the median (Whiskers: 1-99 percentile). Data were analyzed using two-tailed unpaired t-test. (B) miR-128-3p expression in breast cancer patient subtypes luminal A (Lum A; n=353), luminal B (Lum B; n=224), HER2+ (Her2; n=60) and basal (Basal; n=89) of METABRIC expression data set. Horizontal line represents the median (Whiskers: 1-99 percentile). Data were analyzed using two-tailed unpaired t-test. Key: **p < 0.01

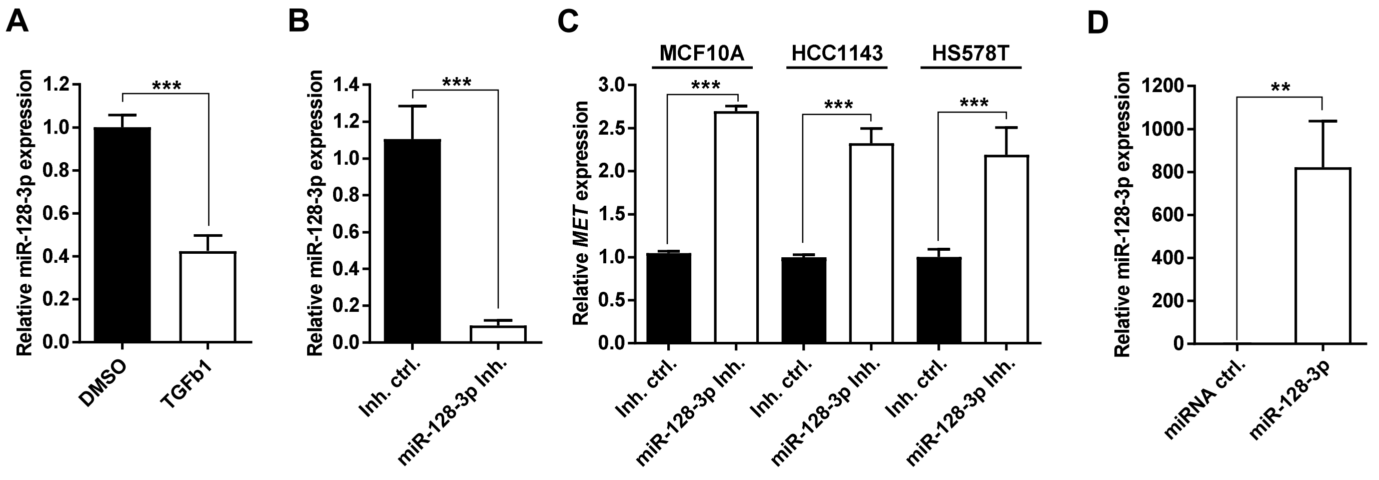

Fig. S11. Regulation of miR-128-3p and *MET* expression by TGFβ1 and miR-128-3p. (A) Quantitative PCR for miR-128-3p expression in MCF10A cell line. Cells were treated with 10 ng/ml TGFβ1 for 48 hours prior analysis. (n=3; Student's unpaired t-test). (B) Quantitative PCR for miR-128-3p expression in MCF10A cell line. Cells were treated with 50nM miR-128-3p inhibitor or inhibitor control for 48 hours prior analysis (n=3; Student's unpaired t-test). (C) Quantitative PCR of *MET* mRNA levels after transfection of MCF10A, HCC1143 and HS578T cells with antisense-miR-128-3p or antisense control (n=3; Student's unpaired t-test). (D) Quantitative PCR for miR-128-3p expression in MCF10A cell line. Cells were treated with 50nM miR-128-3p mimic or miRNA control for 48 hours prior analysis (n=3; Student's unpaired t-test). Data are expressed as mean + SD. Key: **p <0.01, ***p < 0.001.

Table S1: Sequences of siRNAs targeting *MET*, *TGFBR2*, *ETS1* as well as non-targeting siRNA control.

Gene	siRNA no.	Sequence
MET	J-003156-13	GAACUGGUGUCCCGGAUAU
	J-003156-15	GAGCCAGCCUGAAUGAUGA
	J-003156-16	GUAAGUGCCCGAAGUGUAA
TGFBR2	J-003930-06	CAACAACGGUGCAGUCAAG
	J-003930-07	GACGAGAACAUAACACUAG
	J-003930-08	GAAAUGACAUCUCGCUGUA
	J-003930-09	CCAAUAUCCUCGUGAAGAA
ETS1	J-003887-05	AUAGAGAGCUAUGAUAGUU
	J-003887-06	GAAAUGAUGUCUCAAGCAU
	J-003887-07	GUGAAACCAUAUCAAGUUA
	J-003887-08	CAGAAUGACUACUUUGCUA
ON-TARGETplus Non-Targeting siRNA Pool	D-001810-10	UGGUUUACAUGUCGACUAA
		UGGUUUACAUGUUGUGUGA
		UGGUUUACAUGUUUUCUGA
		UGGUUUACAUGUUUUCCUA

Table S2: Sequences and Universal Probe Library (UPL) probe numbers for genes quantified by TaqMan qRT-PCR.

Name	Sequence (5´- 3´)	UPL Probe
TGFBR2_left	caccgcacgttcagaagtc	#43
TGFBR2_right	tggatgggcagtcctattaca	#43
MET_left	tgaaattcatccaaccaaatctt	#31
MET_right	aatagaaaactgacaatgttgagagg	#31
ETS1_left	ccatcatcaagacggaaaaag	#50
ETS1_right	gggacatctgcacattccata	#50
ACTB_left	ccaaccgcgagaagatga	#64
ACTB_right	ccagaggcgtacagggatag	#64
TFRC_left	cccagttgctgtcctgatataga	#61
TFRC_right	ttgagaaaacaatgcaaaatgtg	#61

Table S3: Correlation analysis of TGFBR2 in a data set (Riaz et al) containing mRNA expression of 51 breast cancer cells. Shown are the top 33 correlated genes.

	Gene	correlation coefficient
	TGFBR2	1.0000
1	CAV1	0.9031
2	ITGA3	0.8582
3	DPYD	0.8577
4	MET	0.8523
5	IL15	0.8520
6	CAV2	0.8515
7	LEPREL1	0.8484
8	LY6K	0.8465
9	PTRF	0.8405
10	TGFBI	0.8397
11	RGS20	0.8389
12	CASP4	0.8236
13	PLAU	0.8117
14	OBFC2A	0.8105
15	SRPX2	0.8069
16	EGFR	0.8043
17	ETS1	0.8022
18	S100A2	0.7981
19	HOXA1	0.7943
20	CD44	0.7891
21	C10orf54	0.7863
22	PHLDB2	0.7828
23	NT5E	0.7803
24	SLC16A7	0.7791
25	SP100	0.7787
26	ANXA1	0.7770
27	PLAUR	0.7763
28	ACTN1	0.7758
29	FSTL1	0.7749
30	FOSL1	0.7741
31	IGFBP6	0.7727
32	ST7	0.6523
33	LOXL2	0.6520

Table S4: Transcription factors correlated with (A) *TGFBR2* and (B) *MET* having putative binding site in the MET promoter.

TFs	Correlation with TGFBR2
ETS1	0.802
STAT1	0.590
JUN	0.510
NFKB1	0.423
STAT5B	0.351
SP3	0.303
STAT5A	0.279
cMyc	0.163
SMAD7	0.099
PAX3	0.053
SP1	-0.130

TFs	Correlation with MET
ETS1	0.803
STAT1	0.497
NFKB1	0.448
STAT5B	0.432
JUN	0.398
SP3	0.386
cMyc	0.335
SMAD7	0.146
STAT5A	0.142
PAX3	0.036
SP1	-0.206

Table S5: Correlation analysis of *MET* in a data set (Riaz et al) containing mRNA expression of 51 breast cancer cells. Shown are the top 33 correlated genes.

	Gene	correlation coefficient
	MET	1.0000
1	LEPREL1	0.8988
2	SRPX2	0.8927
3	CAV1	0.8796
4	CAV2	0.8553
5	TGFBR2	0.8523
6	PPARG	0.8393
7	DPYD	0.8333
8	COTL1	0.8289
9	LOC100127972	0.8231
10	OBFC2A	0.8213
11	RGS20	0.8204
12	ITGA3	0.8172
13	HOXA1	0.8148
14	PTRF	0.8144
15	COTL1	0.8078
16	PHLDB2	0.8029
17	ETS1	0.8027
18	PTRF	0.8017
19	IL15	0.7983
20	TAP2	0.7962
21	LYN	0.7927
22	GJC1	0.7919
23	SYTL4	0.7916
24	S100A2	0.7910
25	UPP1	0.7895
26	PTRF	0.7892
27	TGFBI	0.7865
28	FOSL1	0.7857
29	LOXL2	0.7854
30	B3GNT5	0.7810
31	PLAU	0.6556
32	ST7	0.6523
33	LOXL2	0.6520
